# Supplementary material for: A large effective population size for established within-host influenza virus infection
Source: eLife. 2020 Aug 10;9:e56915. doi: 10.7554/eLife.56915 (PMC7431133; doi:10.7554/eLife.56915)
Supplement: Transparent reporting form [file elife-56915-transrepform.docx]

***eLife’s* transparent reporting form**

We encourage authors to provide detailed information *within their submission* to facilitate the interpretation and replication of experiments. Authors can upload supporting documentation to indicate the use of appropriate reporting guidelines for health-related research (see [EQUATOR Network](http://www.equator-network.org/%20)), life science research (see the [BioSharing Information Resource](https://biosharing.org/)), or the [ARRIVE guidelines](http://www.plosbiology.org/article/info:doi/10.1371/journal.pbio.1000412) for reporting work involving animal research. Where applicable, authors should refer to any relevant reporting standards documents in this form.

If you have any questions, please consult our Journal Policies and/or contact us: [editorial@elifesciences.org](mailto:editorial@elifesciences.org).

**Sample-size estimation**

- You should state whether an appropriate sample size was computed when the study was being designed
- You should state the statistical method of sample size computation and any required assumptions
- If no explicit power analysis was used, you should describe how you decided what sample (replicate) size (number) to use

Please outline where this information can be found within the submission (e.g., sections or figure legends), or explain why this information doesn’t apply to your submission:

This is not the kind of study to require sample-size estimation. Sequence data was collected from a clinical case of influenza B infection in a single patient treated by Prof Breuer, samples having been collected in the routine process of treatment of the patient; a clinical case study has been published describing the details of sampling and the role of the data in diagnosing a beneficial effect of favipiravir in clearing infection.

This study therefore arose out of the study of a prior dataset. The number of samples was sufficient for our analysis to work, but the analysis was designed after the collection of data. Our study makes further use of data describing long-term influenza A/H3N2 infection, taken from a previous publication by another group.

**Replicates**

- You should report how often each experiment was performed
- You should include a definition of biological versus technical replication
- The data obtained should be provided and sufficient information should be provided to indicate the number of independent biological and/or technical replicates
- If you encountered any outliers, you should describe how these were handled
- Criteria for exclusion/inclusion of data should be clearly stated
- High-throughput sequence data should be uploaded before submission, with a private link for reviewers provided (these are available from both GEO and ArrayExpress)

Please outline where this information can be found within the submission (e.g., sections or figure legends), or explain why this information doesn’t apply to your submission:

Our analysis involves the comparison of statistics derived from genome sequence data to results from simulations. The analysis of the sequence data was conducted once, no replication being required. In performing simulations 500 replicates of each simulation were initially conducted, however we realized in retrospect that in order to generate a 97.5% confidence interval it would be better to use only 400 of these, giving a neat range from the sorted data by choosing the fifth and three-hundred-and-ninety-sixth samples. Our simulation output files are of the form RunX.dat where X is a random number. For the cases where 500 replicates were performed we chose the first 400 of these in alphabetical order (i.e. where Run13 comes before Run2) to calculate the simulation statistics presented in our manuscript.

**Statistical reporting**

- Statistical analysis methods should be described and justified
- Raw data should be presented in figures whenever informative to do so (typically when N per group is less than 10)
- For each experiment, you should identify the statistical tests used, exact values of N, definitions of center, methods of multiple test correction, and dispersion and precision measures (e.g., mean, median, SD, SEM, confidence intervals; and, for the major substantive results, a measure of effect size (e.g., Pearson's r, Cohen's d)
- Report exact p-values wherever possible alongside the summary statistics and 95% confidence intervals. These should be reported for all key questions and not only when the p-value is less than 0.05.

Please outline where this information can be found within the submission (e.g., sections or figure legends), or explain why this information doesn’t apply to your submission:

Information about all statistical methods is contained within the Methods section.

(For large datasets, or papers with a very large number of statistical tests, you may upload a single table file with tests, Ns, etc., with reference to sections in the manuscript.)

**Group allocation**

- Indicate how samples were allocated into experimental groups (in the case of clinical studies, please specify allocation to treatment method); if randomization was used, please also state if restricted randomization was applied
- Indicate if masking was used during group allocation, data collection and/or data analysis

Please outline where this information can be found within the submission (e.g., sections or figure legends), or explain why this information doesn’t apply to your submission:

Samples were not divided into groups in this way. Phylogenetic and other statistical methods (haplotype reconstruction) were used to identify clades in our influenza B data. All methods are described in the Methods section.

**Additional data files (“source data”)**

- We encourage you to upload relevant additional data files, such as numerical data that are represented as a graph in a figure, or as a summary table
- Where provided, these should be in the most useful format, and they can be uploaded as “Source data” files linked to a main figure or table
- Include model definition files including the full list of parameters used
- Include code used for data analysis (e.g., R, MatLab)
- Avoid stating that data files are “available upon request”

Please indicate the figures or tables for which source data files have been provided:

We have provided source data for Figures 1 and 2, and for the supplementary figures 1S2, 2S1, 2S2, 2S3, 2S4, 2S5 and 2S7. Code and data are provided in a github page, detailed in the Methods section.
